# Soil carbon sequestration potential of planting hedgerows in agricultural landscapes

**DOI:** 10.1016/j.jenvman.2022.114484

**Published:** 2022-04-01

**Authors:** Sofia Biffi, Pippa J. Chapman, Richard P. Grayson, Guy Ziv

**Affiliations:** University of Leeds, School of Geography, Seminary St, Woodhouse, Leeds, LS2 9JT, UK

**Keywords:** Field boundary, Woody linear features, Climate change mitigation, Agri-environment schemes, Grassland, Agroforestry

## Abstract

Realising the carbon (C) sequestration capacity of agricultural soils is needed to reach Paris Climate Agreement goals; thus, quantifying hedgerow planting potential to offset anthropogenic CO_2_ emissions is crucial for accurate climate mitigation modelling. Although being a widespread habitat in England and throughout Europe, the potential of hedgerows to contribute to net-zero targets is unclear. This is the first study to quantify the soil organic carbon (SOC) sequestration rate associated with planting hedgerows. We derived SOC stocks beneath hedgerows based on two estimation methods to assess differences from adjacent intensively managed grassland fields and how these may be affected by sampling depth and hedgerow age, as well as the SOC estimation method used. Twenty-six hedgerows on five dairy farms in Cumbria, England, were classified based on the time since their planting. We measured SOC stocks in 10 cm depth intervals in the top 50 cm of soil beneath hedgerows and in adjacent grassland fields. SOC beneath hedgerows was on average 31.3% higher than in the fields, 3.3% for 2–4 year old hedgerows, 14.4% for 10 year old, 45.2% for 37 year old, and 57.2% for older ones. We show that SOC sequestration rate beneath 37 year old hedgerows was 1.48 Mg C ha^−1^ yr^−1^ in the top 50 cm of soil. If England reaches its goal of a 40% increase in hedgerow length, 6.3 Tg CO_2_ will be stored in the soil over 40 years, annually offsetting 4.7%–6.4% of present-day agricultural CO_2_ emissions. However, the current rate of planting funded by agri-environment schemes, which today reaches only 0.02% of emissions, is too slow. Private-sector payments for ecosystem services initiatives (e.g., ‘Milk Plan’) show much higher rates of planting and are needed alongside agri-environment schemes to ensure hedgerow planting contributes to net-zero targets.

## Highlights

• On average, 31% more carbon was stored in soil beneath hedgerows than grassland.
• Soil organic carbon (SOC) sequestration rate beneath hedgerows is 1.48 Mg C ha^−1^ yr^−1^.
• Increasing hedgerows in England by 40% will sequester and store 6.3 Mg of CO_2_ as SOC.
• Private and public schemes must combine efforts to aid climate change mitigation.

## 1 Introduction

Soil degradation through climate change, enhanced crop output, accelerated erosion, and intensive agricultural practices have resulted in a decline in SOC stocks and created a large C debt in soils of ∼ 40–90 Pg C (Smith, 2004). Thus, SOC sequestration in agricultural soils is a promising route towards climate change mitigation (Lal, 2003, 2004; Smith et al., 2008; Minasny et al., 2017), with the additional benefits of improving soil health and resilience (Lal, 2006).

Hedgerows are woody linear features common in farmed landscapes around the world, sometimes dating back thousands of years (Baudry et al., 2000), that have undergone strong declines in recent decades. They are human-created systems of closely spaced shrubs and trees that delineate field boundaries and provided shelter for crops and livestock. In the UK, hedgerows are a defining feature of agricultural landscapes (Oreszczyn and Lane, 2000) managed by regular trimming every one to three years and occasional structural restoration (>40 years) to improve their windbreak and livestock holding function (Axe et al., 2017). Thus, British hedgerows are generally low—often less than 2 m—continuous lines of trimmed bushes dominated by hawthorn (*Crataegus monogyna* Jacq.) and blackthorn (*Prunus spinosa* L., Barr and Gillespie, 2000), differing from other regions where hedgerows are frequently much taller, of different species composition, and less subject to periodic management regimes (e.g. Montgomery et al., 2020; Litza and Diekmann, 2020; Van Den Berge et al., 2021; Viaud and Kunnemann, 2021). Hedgerows have declined markedly in many countries since the mid-20th century (e.g. Baltensperger, 1987; Barr and Gillespie, 2000) and over a million km of hedgerows have been lost in England and Wales since 1945 (O'Connell et al., 2004). Today, in many countries including the UK, legislation protects hedgerows and impedes their removal (Baudry et al., 2000; Oreszczyn and Lane, 2000); however, in some areas hedgerows continue to decline (Kristensen et al., 2016; Arnaiz-Schmitz et al., 2018). In England, there were ∼400,000 km of managed hedgerows in 2007, 21% less than in 1984 (Carey et al., 2007). Thus, over this period English hedgerows declined on average by 4739 km yr^−1^.

The provision of ecosystem services by hedgerows has fuelled their inclusion in agri-environment schemes (AES). Aside from their historical role of crop protection and livestock enclosure, hedgerows have been shown to provide significant above-ground biodiversity benefits within farmed landscapes (Heath et al., 2017; Kremen et al., 2018; Litza and Diekmann, 2019). More recently, their role in providing wider ecosystem service benefits, such as nutrient interception and protection of surface water quality, flood and drought mitigation, and climate change mitigation has been investigated (Marshall and Moonen, 2002; Bianchi et al., 2006; Benhamou et al., 2013; Van Vooren et al., 2018; Graham et al., 2018; Holden et al., 2019; Wallace et al., 2021; Weninger et al., 2021). Therefore, hedgerow planting and management has been encouraged through public AES, such as the Countryside Stewardship, Environmental Stewardship, and Sustainable Farming Incentives in the UK (DEFRA, 2020, 2021; Natural England and Rural Payments Agency, 2021), as well as private sector initiatives (Tipper and Elliott, 2018; CISL, 2018; Elliot, 2020), where it is increasingly acknowledged that degradation of agricultural ecosystems can lead to operational risks (Seddon et al., 2020).

Determining the contribution of planting hedgerows to atmospheric C sequestration and SOC storage in agricultural landscapes is urgently needed for climate mitigation modelling. Hedgerows may play a part in climate change mitigation (Climate Change Committee, 2018, 2019), as they can sequester and store C in their biomass, as well as in the soil beneath them (Peichl et al., 2006; Schoeneberger et al., 2012; D'Acunto et al., 2014; Kay et al., 2019; Ford et al., 2019; Viaud and Kunnemann, 2021). Hedgerow management has been shown to have a large impact on hedgerow aboveground C storage (Axe et al., 2017) and in the UK, where management is frequent and species diversity is low (Lange et al., 2015; Carey et al., 2007), SOC storage is likely to contribute significantly to hedgerow storage potential. However, the change in SOC stock as a result of planting hedgerows, albeit acknowledged, is not well quantified (Follain et al., 2007; Thiel et al., 2015; Wolton et al., 2014). This has led to contrasting messages about the role hedgerows can play in climate change mitigation. For example, while the Climate Change Committee call for a 40% increase in hedgerow length in the UK to meet “net-zero” targets (Climate Change Committee, 2018, 2019; 2020b), there have also been estimates of no contribution from hedgerows towards reaching the same goal (Thomson et al., 2018). A few previous studies have quantified SOC stocks close to hedgerows (e.g. Ford et al., 2019; Van Den Berge et al., 2021), and, currently, land managers are encouraged to increase hedgerows height and width to increase aboveground biomass C stock, and to plant new hedgerows along field boundaries to increase SOC stock over time (Wolton et al., 2014; Axe et al., 2017; Gregg et al., 2021). However, the rate at which hedgerows accumulate and store C in their biomass and soil beneath them has yet to be quantified, meaning it is hard to predict their contribution to climate change mitigation. In addition, there is little information on the rates at which they are being planted and how this compares to the rate at which they have declined.

Accurate rates of CO_2_ sequestration by soil beneath hedgerows are limited by lack of information regarding SOC stocks beneath managed hedgerows and how factors such as soil depth and hedgerow age may affect them (Wolton et al., 2014), as well as how SOC stock beneath hedgerows may compare to those in adjacent agricultural fields (Ford et al., 2021; Viaud and Kunnemann, 2021). The SOC stock beneath hedgerows will depend on a number of factors, such as the maturation stage of the hedgerow, previous land use, the soil physical and chemical properties at the time of planting, and the depth of sampling (Laganière et al., 2010). The age of hedgerows (i.e., years since planting) is likely to influence the SOC stock due to changes in the quantity, quality, and rate of C inputs from fine root and hyphal turnover, exudation, and accumulation of leaf litter over time as the plants grow and mature (Godbold et al., 2006; Orwin et al., 2011; Yan et al., 2018) over the course of several decades (Falloon et al., 2004). Moreover, soil type is likely to influence the rate of SOC accumulation as soil texture and moisture regime have a large control on microbial activity and, thus, decomposition rates of soil organic matter as well as mineral adsorption of SOC (Wiesmeier et al., 2013, 2019). Soil chemistry also plays an important role as, for example, pH can affect SOC mineralisation rates (Pietri and Brookes, 2008). Finally, SOC is not uniformly distributed down the soil profile, due to differential distribution of roots and microbial populations with depth (Jobbagy and Jackson, 2001; Angst et al., 2018), thus SOC stocks depend on soil sampling depth.

Our study is based on comparative observations of SOC stocks beneath hedgerows on five farms participating in the Nestlé-First Milk ‘Milk Plan’ sustainable supply chain initiative in Cumbria, UK. Milk Plan offers a premium payment to dairy farmers who adopt environmentally friendly practices on their land, such as tree and hedgerow planting. Our main aim was to determine SOC stocks beneath hedgerows of different ages and in adjacent grassland fields to estimate the C sequestration rate of soil beneath hedgerows. We quantified SOC stocks at 10 cm intervals from 0 to 50 cm depth under hedgerows of known age classes and hypothesized that *i*) SOC stock beneath hedgerows would be higher than in the adjacent improved grassland fields and *ii*) SOC stock would increase with hedgerow age (years since planting). We evaluated differences in SOC storage estimates by calculating SOC stocks using fixed depth (FD) and equivalent soil mass (ESM) methods. A secondary aim was to use the SOC sequestration rate determined in this study and the hedgerow planting rates within the Milk Plan and AES in England to estimate (1) the amount of SOC sequestered by soil by hedgerow planting initiatives in the Eden Valley, Cumbria, and across the whole of England and (2) the SOC sequestration potential of increasing existing hedgerows length by 40%, as advised by the Climate Change Committee mitigation models, and how long it would take to be achieved given current planting rates.

## 2 Methods

### 2.1 Study sites

The study area encompassed five dairy farms located within the county of Cumbria, Northwest England, in the Eden Valley, which separates the Cumbrian Mountains of the Lake District from the Northern Pennines. The valley was formed by rifting in the Permian period and contains Permian and Triassic sediments that include aeolian sandstone, mudstone and siltstone which lie unconformably over Carboniferous limestone (Allen et al., 2010). The farms were all taking part in the Milk Plan, which offers options similar to those in public AES. For example, the hedgerow planting option follows the same guidelines as within the Countryside Stewardship scheme (option ‘BN11 Planting new hedges’, Rural Payments Agency and Natural England, 2015). The Koppen climate classification of the region is temperate oceanic (Beck et al., 2018). Average rainfall, temperature, and elevation for each farm are shown in Table 1; average slope among fields was 1.7° (range 0.2–8.6°). The soil on the farms fell into two main groups, freely draining, slightly acid, loamy cambisols and slowly permeable, seasonally wet, slightly acid but base-rich, loamy and clayey stagnosols (WRB, 2015; Cranfield University, 2018). These soil types have been found to contain similar size SOC stocks to 80 cm depth under woodland (Morison, 2012) and they occur extensively across England, 15.5% and 19.9% respectively, with the cambisols suitable for a range of crops and characterized by a long grazing season when under grass production. Stagnosols are mostly suited to grass production for dairy or beef, with some cereal production often used for feed. The Agricultural Land Class of the area is grade 3 (‘good to moderate quality agricultural land’, Natural England, 2010). The sampled fields were in permanent pasture (83%) or leys with the occasional arable crop (19%). The fields were intensively managed and predominantly classified as MG7 (‘*Lolium perenne* L. reseeded grassland’, Rodwell, 1998), and most were cut annually for silage.

**Table 1:** Average rainfall and temperature (Met Office, 2020), altitude, number of hedgerows, and soil type of each farm. C = cambisol, S = stagnosol.

Farm	Temp (°C)	Rain (mm)	Altitude (m)	Bedrock	Soil type	Hedges sampled
1	8.9	1054	181	Sandstone	C	2–4 years (2); 10 years (4); Old (1)
2	9.6	985	19	Sandstone	S	Old (1)
3	8.5	1152	175	Sandstone	C	2–4 years (1); Old (1)
4	8.6	1194	108	Limestone, sandstone, siltstone, mudstone	S	10 years (1); Old (2)
5	8.6	1074	191	Limestone, mudstone, siltstone, sandstone	S	2–4 years (2); 10 years (1); 37 years (8); Old (2)

### 2.2 Hedgerow characteristics

**Fig. 1:**
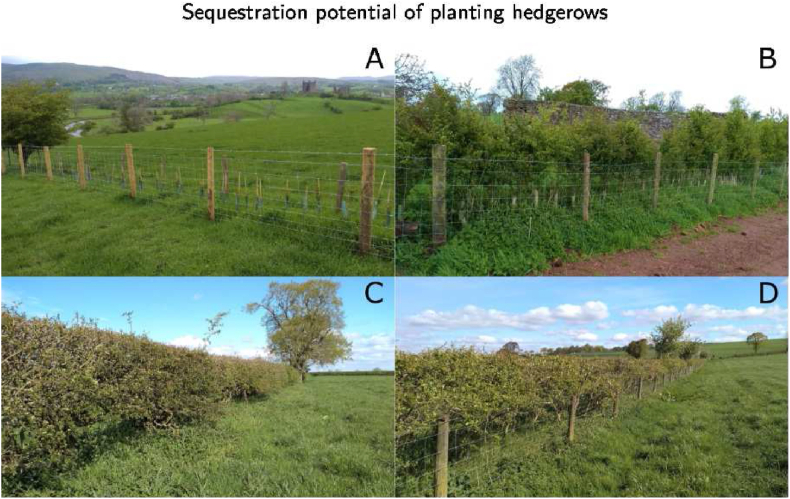
Example of hedgerows used in this study based on their age category: 2–4 year old (A), 10 year old (B), 37 year old (C), Old (D) hedgerows.

Across the five farms, 32 hedgerows were selected and grouped into four age categories: (1) ‘2–4 year old’ if they were planted after 2017 as part of Milk Plan agreements, (2) ‘10 year old’ if they were planted between 2016 and 2010, (3) ‘Old’ if they were planted before 2010, and (4) ‘37 year old’ for eight old hedgerows for which the exact year of planting was known (Fig. 1). The Old hedgerow category potentially included a wide range of ages, from tens to hundreds of years, and for most of the hedgerows the exact year of planting was not known. The species composition of the hedgerows was typical of Cumbria (Cumbria Biodiversity Data Centre, 2010), with a strong predominance of hawthorn (*Crataegus monogyna* Jacq. 70%) and blackthorn (*Prunus spinosa* L., 15%), and presence of hazel (*Corylus avellana* L., 2%), elder (*Sambucus nigra* L., 1%), holly (*Ilex aquifolium* L., <1%), and dog-rose (*Rosa canina* L., <1%). Hawthorn and blackthorn are the predominant woody species in hedgerows across England and Wales (Barr and Gillespie, 2000; Carey et al., 2007). Fully grown hedgerows (37 year old and Old ones) were managed via trimming using a tractor mounted flail mower every one to two years, with trimmed residues left to decompose. Most hedgerows were fenced, as shown in Fig. 1. On average, hedgerows older than ten years were 1.75 m tall and 1.70 m wide, while 2–4 year old ones were 1.27 m tall, and 0.76 m wide. All hedgerows, apart from two 37 year old hedges, were planted where according to 18th century mapping hedgerows had been historically present (EDINA, 2010).

### 2.3 Soil sampling and analysis

We used a space-for-time substitution approach, where a point-in-time sample from under hedgerows of different ages was compared against an assumed business-as-usual baseline from an adjacent agricultural field. Thus, we compared SOC stocks between pairs of samples taken at a single point in time so many years after a change in land use (improved grass to hedgerow, in this case) had occurred at one of the sites. This approach assumes that the field and hedgerow sites were the same prior to the change in land use (i.e. in terms of soil type, climate, land use, productivity). Therefore, we excluded from further analysis paired samples that were not located on the same soil type. Table 1 shows the final number of hedgerows included within each age category.

All soil samples were collected between May and November 2019. For all pairs of samples, the sampling point under the hedgerow was randomly selected (avoiding gateways, tracks, and gaps) and the point in the adjacent field was at 16 m perpendicular from the hedgerow. Thus, 26 paired samples were collected, resulting in 52 sampling locations. At each location, a 5 cm diameter ring corer (Eijkelkamp, Holland) was used to take intact 100 cm^3^ soil cores at 2–7, 12–17, 22–27, 32–37, and 42–47 cm, representing the layers 0–10, 10–20, 20–30, 30–40, and 40–50 cm, respectively, for the determination of bulk density and moisture content. At each sampling location two grab soil samples were also collected from each depth for the determination of soil organic matter (SOM) and SOC content. Grab soil samples were also taken from three depths only (0–10, 10–20, 30–40 cm) for the determination of soil pH. On return to the laboratory the ring samples were oven dried at 105 °C for 48 h and sieved to <2 mm diameter for the determination of soil moisture content (g g^−1^) and bulk density (BD g cm^−3^). Gravel and roots >2 mm were removed, and their masses recorded. Bulk density was calculated as the difference between the total sample mass and the mass of gravel and roots, divided by the sample volume (Poeplau et al., 2017). One grab sample was sieved to 5 mm to homogenise the soil, oven dried at 105 °C, and then placed in a furnace for 16 h at 550 °C for the determination of SOM content (g cm^−2^) via the loss on ignition method. The second grab soil samples were air-dried at 40 °C (<2 mm), and then milled to a fine powder using a ball mill (Fritsch Pulverisette agate, Fritsch, Germany) to determine SOC (g kg^−1^). Inorganic C was removed from soil samples by reaction with acid. Samples of <100μm milled soil were placed into 9 × 5 mm silver capsules and 30 μL of 15% HCl was slowly added. The samples were left to react and settle for 24 h and oven dried for 2 h at 80 °C before being analysed for C using an Elemental Vario EL cube (Elementar Analysensysteme GmbH, Germany). Soil pH was measured using 10 g air-dried soil (<2 mm) with 25 ml of distilled water in a 1:2.5 dilution method (Rowell, 2014) and a pH meter (pH700 benchtop meter, Oakton) calibrated with pH 4 and 7 buffers and checked every ten samples.

The SOC stock (Mg C ha^−1^) was estimated for each location using two methods to allow for comparison with previous studies. Firstly, using the traditional and widely used fixed depth (FD) method of multiplying SOC concentration by bulk density to a fixed soil depth as:

(1) $${SOC}_{FD}=\overset{n}{\underset{i=1}{\sum }}{SOC}_{\text{con}i}\times {BD}_{i}\times t_{i}\;,$$

where SOC_*FD*_ is the SOC stock of the investigated soil profile to a certain depth *n*, SOC_con*i*_ is the SOC (%), BD_*i*_ is the bulk density (g cm^−3^), and *t*_*i*_ is the respective thickness of the soil layer sampled. We present SOC stocks for 0–30 cm depth (n = 3) and 0–50 cm depth (n = 5).

Secondly, SOC stocks were calculated using the equivalent soil mass (ESM) correction (Wendt and Hauser, 2013; von Haden et al., 2020). To do so, we relied on the cumulative coordinate approach (Gifford and Roderick, 2003; Wuest, 2009). We used model fitting to adjust SOC stocks to the reference cumulative mineral soil mass in the adjacent field, assuming exponentially decaying SOC through the soil profile (Rovira et al., 2015; Murphy et al., 2019, Appendix A), as:

(2) $${SOC}_{ESMi}=f\left(CMM_{\mathrm{fi}eld,i}\right)=a\times \left(1-exp\left(-b\times {CMM}_{\mathrm{fi}eld,i}\right)\right)\;,$$

where for each sampling site we define a function *f* associating cumulative SOC_*con*_ to cumulative mineral soil mass (CMM). The mineral soil mass of the samples (g cm^−2^) was calculated as the difference between soil mass of the samples and the SOM content (g cm^−2^). The function *f* is obtained by interpolating the hedgerow measurements via non-linear least squares (nls) to fit the curve *f*(*x*) = *a* × (1 − *exp*(−*b* × *x*)). CMM_*field*,*i*_ (g cm^−2^) is the in-field CMM at depth *i*. We compute estimates for SOC_*ESM*_ (g cm^−2^) at depth *i* by evaluating *f* at CMM_*field*,*i*_ and present hedgerow SOC_*ESM*_ for equivalent soil mass in the field at 0–30 cm and 0–50 cm depth. Moreover, we calculated the additional SOC_*ESM*_ stock accumulated over time as a result of planting hedgerows as the difference between SOC_*ESM*_ stocks in the hedgerows and fields.

Finally, it was possible to determine an average annual soil sequestration (Mg C ha^−1^ yr^−1^) rate for each hedgerow age category as:

(3) $$SOCseq.=\frac{{SOC}_{ESM}hedgerow-{SOC}_{ESM}\mathrm{fi}eld}{yearssinceplanting}\;,$$

where years since planting was assumed to be 3, 10, 37, and 50 years. Fifty years were assumed to be the time for the soil to reach a new equilibrium and thus stop sequestering additional SOC beneath Old hedgerows (Falloon et al., 2004; Drexler et al., 2021). The sequestration rate was also reported by length of hedgerow (Mg C km^−1^yr^−1^), assuming a representative width of 1.5 m (Falloon et al., 2004; Carey et al., 2007; Axe et al., 2017) and as an annual CO_2_ sequestration rate (Mg CO_2_ km^−1^ yr^−1^) by multiplying Mg C by the ratio of molecular weight of CO_2_ to that of C (ratio = 3.67).

### 2.4 Data analysis

Differences in pH, bulk density, root and gravel content, SOC_*con*_ (g kg^−1^) content, and SOC stock between grassland fields and hedgerows of different age classes and soil types were investigated using ANOVAs or non-parametric Kruskal-Wallis rank test when the data distribution did not meet the assumption of normality. In case of significant differences, these were followed by pairwise t-tests or Wilcoxon signed rank tests for post-hoc pairwise comparisons with Benjamini-Hochberg false discovery rate-corrected P-values. Associations between SOC stock, hedgerow age, and other explanatory variables, was investigated using linear models, with differences considered significant at P <0.05. Separate linear models were fitted for SOC_*FD*_ and SOC_*ESM*_ stock across all sampling depths. Hedgerow age category, depth of soil sample, and soil type (cambisol or stagnosol) were included as categorical predictors, as well as environmental covariates of average rainfall (mm) and average temperature (°C) in 2009–2019. Predictors were scaled for comparability of effect size. Model assumptions were checked with residual plotting and assumption were met. All analyses were conducted in R (R. Core Team, 2021).

#### 2.4.1 Up-scaling of results to estimate C uptake by soils as a result of planting hedgerows

Soil disturbance associated with planting a new hedgerow can result in compaction of the soil and displacement of the organic layer to deeper within the soil profile (Laganière et al., 2010). This may result in higher estimates of SOC stock and sequestration rate in the initial years after planting, which, together with SOC stock saturation as soils reach a new equilibrium (Caruso et al., 2018), could bias the interpretations of the long-term effects of hedgerows planting on SOC stocks and SOC sequestration rates. Thus, to assess the impact of hedgerow planting in the long term, we considered the sequestration rate of the oldest hedgerows of known age (i.e. 37 years old).

The soil C sequestration rate determined in this study was used to estimate the amount of SOC sequestered by hedgerows planted under hedge-planting schemes in the Eden Valley, Cumbria, and across England by 2050. In the Eden Valley, the upscaling area was limited to cambisols and stagnosols below 230 m and 8.6° slope for consistency with the characteristics of the sites sampled in our study. The SOC stock was calculated for the length of all hedgerows planted within AES (option PH ‘Hedgerow planting new hedges’ in the Environmental Stewardship and BN11 ‘Planting new hedges’ in the Countryside Stewardship, Natural England, 2020a,b) 2004–2019, and as part of Milk Plan's hedgerow planting initiative in 2017–2020.

Across England, we estimated the amount of SOC that will be sequestered by 2050 beneath hedgerows planted along boundaries of improved grassland and arable fields within all AES in 2004–2019 as:

(4) $${SOC}_{AES}=\overset{N}{\underset{i=1}{\sum }}SOCseq.\times {len}_{i}\times {yrs}_{i}\;,$$

where SOC_*AES*_ is the SOC stock beneath all hedgerows planted within a total of *N* AES agreements, *i* ranges over the AES agreements, len_*i*_ is the length of hedgerow planted within agreement *i*, and yrs_*i*_ is the number of years between planting of hedgerows in agreement *i* and 2050. Agreements were selected based on landcover classification of the UKCEH Land Cover Map (LMC2018) at 25 m resolution.

Finally, we estimated ΔSOC_*CCC*_, the additional SOC storage 40 years after increasing the length of existing hedgerows across the country by 40% as:

(5) $$\Delta {SOC}_{CCC}=SOCseq.\times {len}_{CCC}\times {yrs}_{CCC}=SOCseq.\times 0.4\times {len}_{England}\times 40\;,$$

where the length of existing hedgerows (len_*England*_) was calculated as the estimated length of well-maintained hedgerows and tree lines in 2007 (Carey et al., 2007), plus hedgerows planted by AES around improved grassland and arable fields in 2004–2019 (Natural England, 2020a, 2020b).

It should be noted that SOC sequestration varies depending on soil type and climate and our estimate of SOC sequestration is associated with hedgerow planting around grassland fields in a cool, wet climate. Therefore, the figures of total hedgerow planting and SOC sequestration potential presented here are indicative and should be considered as such. Moreover, the SOC sequestration rates determined in this study refer to hedgerows predominantly consisting of hawthorn and blackthorn, which are typical in the UK (Carey et al., 2007).We do not discuss how these rates may vary beneath hedgerows dominated by different species or with management regime.

## 3 Results

### 3.1 Soil characteristics beneath hedgerows of different ages in comparison to grassland fields

Soil beneath hedgerows differed in pH, bulk density, root content and SOC_*con*_ content from adjacent fields, while moisture content and gravel content volume was not significantly different across them (Table 2). Particularly, soil pH was higher in fields and beneath 37 year old hedgerows than in the other hedgerow age classes while, in contrast, bulk density was higher in fields and 2–4 year old hedges. SOC_*con*_ was significantly higher beneath Old and 37 year old hedgerows and root content was higher beneath older hedgerows compared to adjacent fields and younger hedgerows at 0–50 cm depth.

**Table 2:** Average (and 95% confidence intervals) of pH, bulk density corrected by gravel and root content, moisture content, root content, and gravel content volume of the soils collected in this study. Different letters indicate statistically significant differences (P <0.05). pH values were obtained only for 0–10, 10–20, and 30–40 cm depth intervals.

Treatment	Depth (cm)	pH	Sig		BD (g cm^3^)*	Sig		Moisture (g g^−1^)*	Sig		Roots (g)	Sig.		Gravel (g)	Sig		SOC_*con*_ (g kg^−1^)	Sig	
Field	0–50	5.9 (5.8–6.1)	a	a	1.40 (1.38–1.42)	a	a	0.25 (0.24–0.26)	ns	ns	0.07 (0.04–0.1)	ns	b	9.3 (2.3–16.2)	ns	ns	19.59 (18.51–20.67)	b	b
Hedgerow																			
*all ages*		5.2 (4.8–5.5)	b		1.19 (1.17–1.22)	b		0.25 (0.24–0.27)	ns		0.66 (0.44–0.89)	ns		10.3 (1.7–18.9)	ns		29.97 (27.82–32.11)	a	
2–4 years		5.1 (4.3–5.9)		b	1.33 (1.29–1.37)		ab	0.22 (0.18–0.25)		ns	0.29 (0.05–0.53)		ab	13.5(-20.2–47.1)		ns	22.22 (19.66–24.78)		ab
10 years		5.2 (4.7–5.7)		b	1.18 (1.13–1.24)		bc	0.21 (0.18–0.23)		ns	0.22 (0.03–0.4)		ab	4.2 (-6.6–14.9)		ns	25.59 (22.57–28.61)		ab
37 years		5.8 (5.1–6.5)		a	1.22 (1.19–1.24)		b	0.30 (0.27–0.32)		ns	0.91 (0.3–1.51)		ab	4.7 (-2.6–12.0)		ns	30.03 (28.14–31.92)		a
Old		4.8 (4–5.5)		b	1.06 (1.00–1.13)		c	0.27 (0.24–0.29)		ns	1.04 (0.63–1.46)		a	19.7 (-9.9–49.3)		ns	40.71 (34.6–46.83)		a
Field	0–30	5.8 (5.7–6)	a	a	1.30 (1.26–1.33)	a	a	0.27 (0.25–0.29)	ns	ns	0.07 (0.03–0.11)	ns	ns	5.3 (0.2–10.3)	ns	ns	26.23 (24.61–27.84)	b	b
Hedgerow																			
*all ages*		5.1 (4.8–5.4)	b		1.08 (1.04–1.12)	b		0.26 (0.24–0.28)	ns		0.34 (0.17–0.52)	ns		5.7 (0.4–11.0)	ns		37.76 (34.23–41.29)	a	
2–4 years		5.2 (4.4–5.9)		b	1.23 (1.15–1.3)		ab	0.23 (0.18–0.29)		ns	0.29 (-0.04–0.62)		ns	9.0 (-13.8–31.8)		ns	29.78 (24.45–35.12)		ab
10 years		5.1 (4.6–5.6)		b	1.04 (0.95–1.14)		bc	0.19 (0.16–0.22)		ns	0.15 (-0.02–0.33)		ns	3.5 (-5.5–12.6)		ns	32.63 (27.09–38.16)		ab
37 years		5.6 (4.9–6.4)		a	1.16 (1.12–1.19)		ab	0.32 (0.29–0.35)		ns	0.42 (-0.06–0.9)		ns	1.5 (-0.8–3.9)		ns	37.68 (34.21–41.14)		a
Old		4.8 (4–5.5)		b	0.92 (0.83–1.00)		c	0.27 (0.23–0.31)		ns	0.46 (0.19–0.74)		ns	10.0 (-7.6–27.5)		ns	47.95 (37.62–58.27)		a

### 3.2 SOC stocks and sequestration rates for hedges of different ages

The different methods used to determine SOC stock yielded different results (Table 3). Hedgerows showed higher SOC_*ESM*_ stock when compared to adjacent fields, a difference that increased with depth of sampling. At 0–50 cm depth, hedgerows older than 10 years had significantly higher SOC_*ESM*_ stock than adjacent fields. The SOC stored beneath the 2–4 year old hedgerows did not differ significantly from that stored in the fields or beneath Old hedgerows but was significantly lower than 37 years old hedgerows. This could be explained by the wide range of hedgerow ages within the Old hedgerow category, some of which may have been planted <37 years prior. Significant differences in SOC_*FD*_ emerged only between the 37 years old hedgerows and adjacent fields.

**Table 3:** Average SOC stock (and 95% confidence intervals) in Mg C ha^−1^ according to the two estimation methods used in the study. SOC_*FD*_ = fixed depth SOC stock estimate; SOC_*ESM*_ = equivalent soil mass SOC stock estimate. Different letters indicate statistically significant differences (P < 0.05).

Treatment	Depth (cm)	SOC_*FD*_	Sig		SOC_*ESM*_	Sig	
Field	0–50	124.9 (112.8–136.9)	b	b	124.9 (112.8–136.9)	b	c
Hedgerow							
*all ages*		158.4 (139.4–177.5)	a		164.0 (144.2–183.8)	a	
2–4 years		130.8 (98.7–163)		ab	129.0 (100.2–157.8)		bc
10 years		131.7 (112.1–151.3)		b	142.8.0 (124.1–161.5)		abc
37 years		175.3 (148.7–201.8)		a	181.3 (153.2–209.5)		a
Old		185.7 (111.8–259.7)		ab	196.3 (129.3–263.3)		ab
Field	0–30	97.3 (89.3–105.3)	b	b	97.3 (89.3–105.3)	b	b
Hedgerow							
*all ages*		111.2 (98.2–124.1)	a		126.8 (111.9–141.7)	a	
2–4 years		99.4 (69.3–129.4)		ab	105.3 (71.8–138.8)		ab
10 years		91.1 (71.5–110.6)		ab	110.5 (92.1–128.8)		ab
37 years		127.0 (105.7–148.2)		a	135.7 (113.1–158.3)		a
Old		118.7 (78.2–159.2)		ab	146.0 (96.1–195.9)		ab

**Fig. 2:**
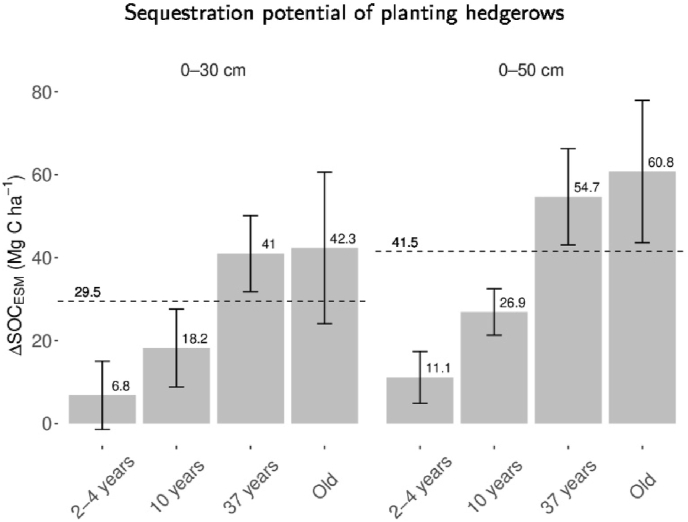
Error bars, mean ± St.Error of additional SOC_*ESM*_ stock (ΔSOC_*ESM*_) in hedgerows of different ages in comparison to adjacent fields at 0–30 cm and 0–50 cm depth of the soil profile. Dashed lines represent the average additional SOC_*ESM*_ stock across all hedgerows in comparison to adjacent fields for 0–30 cm and 0–50 cm sampling depths.

The size of SOC_*ESM*_ stock increased, relative to the adjacent field, with hedgerow age. On average the SOC_*ESM*_ stock beneath hedgerows was 31.3% higher than in the adjacent grassland fields, with 2–4 year old hedgerows showing SOC_*ESM*_ 3.3% higher than fields, 10 year old ones 14.4%, 37 year old ones 45.2%, and Old ones 57.2%. This pattern was reflected by average ΔSOC_*ESM*_ values, which increased across the hedgerow age classes, indicating a progressive build-up of SOC in the soil beneath hedgerows in time (Fig. 2). ΔSOC_*ESM*_ increased by 446% between 2 and 4 year old hedgerows and Old ones.

Model estimates showed that SOC stocks in the first 50 cm of soil were higher in mature hedgerows within the 37 year old and Old age categories than in the fields when accounting for environmental covariates, soil depth, and soil type (Appendix B). The SOC_*FD*_ model (R^2^ = 0.57) showed significantly higher SOC stocks beneath all hedgerow ages compared to fields. ESM estimates, instead, indicated that SOC_*ESM*_ stocks were higher than fields in hedgerows of 10 years and older, with larger effect sizes associated with older hedgerows than newer ones (R^2^ = 0.57). In both models, the depth of the soil sample was negatively associated with SOC stock, with samples closer to the surface having higher SOC stock than deeper ones, as would be expected.

Soil type had a significant effect on SOC stocks and other soil characteristics. Across fields and hedgerows, SOC_*ESM*_ stocks were significantly higher in stagnosols 318.5 (282.6–354.4) than cambisols 239.8 (199.7–279.9) Mg C ha^−1^, *χ*^2^ = 12.4, P <0.001). Moisture content was also significantly higher in stagnosls 0.29 (0.27–0.31) g g^−1^ than cambisols 0.16 (0.14–0.20) g g^−1^, *χ*^2^ = 30.5, P <0.001. Cambisols, instead, had significantly higher bulk density 1.4 (1.2–1.5) g cm^−3^ than stagnosols 1.2 (1.2–1.3) g cm^−3^, *χ*^2^ = 5.3, P = 0.021.

Table 4 shows the SOC_*ESM*_ sequestration rate for two set depth for each of the age categories. Our results show that over time the sequestration rate declines from 3.71 Mg C ha^−1^ yr^−1^ for the 2–4 year old hedgerows to 1.48 Mg C ha^−1^ yr^−1^ for the 37 year old hedgerows, and that after 37 years the sequestration rate declines gradually.

**Table 4:** Estimated annual SOC_*ESM*_ sequestration rates beneath hedgerows assuming a hedgerow width of 1.5 m at 0–50 and 0–30 cm depth of the soil profile. Figures in bold indicate estimates from hedgerows for which the exact year of planting was known.

Depth(cm)	Hedgerow age	Mg C ha^−1^ yr^−1^	Mg C km^−1^ yr^−1^	Mg CO_2_ km^−1^ yr^−1^
0–50	2–4 years	3.71 (-2.08–9.5)	0.56 (-0.31–1.42)	2.04 (-1.14–5.23)
	10 years	2.69 (1.25–4.13)	0.40 (0.19–0.62)	1.48 (0.69–2.28)
	37 years	**1.48** **(0.74**–**2.22)**	**0.22** **(0.11**–**0.33)**	**0.81** **(0.41**–**1.22)**
	Old	1.22 (0.38–2.06)	0.18 (0.06–0.31)	0.67 (0.21–1.13)
0–30	2–4 years	2.28 (-5.33–9.88)	0.34 (-0.8–1.48)	1.25 (-2.93–5.44)
	10 years	1.82 (-0.59–4.24)	0.27 (-0.09–0.64)	1.00 (-0.33–2.33)
	37 years	**1.11** **(0.52**–**1.69)**	**0.17** **(0.08**–**0.25)**	**0.61** **(0.29**–**0.93)**
	Old	0.85 (-0.05–1.74)	0.13 (-0.01–0.26)	0.47 (-0.03–0.96)

### 3.3 Scaling-up: SOC sequestration of hedgerow planting in the Eden valley and across England

Thirty-two farmers participated in the Milk Plan over four years (2017–2020), of which 27 chose the hedgerow planting option, which resulted in the planting of 12.9 km of hedgerows. This length is comparable to the 13.4 km of hedgerows planted around improved grassland and arable fields across twenty-six farms under Countryside and Environmental stewardship AES in the Eden Valley over the period 2004–2019. Thus, the rate of hedgerow planting in the Milk Plan scheme, of 3.2 (2.5–3.9) km yr^−1^, was nearly four times that planted under the AES, which was 0.8 (0.1–1.5) km yr^−1^. Using our SOC_*ESM*_ sequestration rate of 1.48 Mg C ha^−1^ yr^−1^ and an assumed hedgerow width of 1.5 m, we estimated that by 2050 an additional 90 Mg C (330 Mg CO_2_) will be stored in the top 50 cm of soil beneath hedgerows planted within the Milk Plan scheme and an additional 100 Mg C (366 Mg CO_2_) will be stored in the top 50 cm of soil beneath hedgerows planted under AES in the Eden Valley.

Across England a total of 1684 km of hedgerows were planted under AES around improved grassland and arable fields between 2004 and 2019 (Natural England, 2020a, 2020b), at an annual planting rate of 105.2 (42.9–157.5) km yr^−1^. However, it should be noted that this planting rate increased over time, by on average 116 (3–229)% each year, with the highest planting rate, 424.7 km, achieved in 2019. Approximately half of these hedgerows, 754 km, were planted around improved grassland fields. Using our SOC_*ESM*_ sequestration rate of 1.48 Mg C ha^−1^ yr^−1^ and a hedgerow width of 1.5 m, we estimate that by 2050 an additional 12,809 Mg C (47,010 Mg CO_2_) will be sequestered in the soil beneath AES hedgerows planted around improved grassland fields.

Table 5 shows the potential amount of CO_2_ that could be sequestered by agricultural soils in England as a result of a 40% increase in existing hedgerow length which has been proposed by the Climate Change Committee to help reach UK net-zero carbon targets by 2050. The difference between the length of existing hedgerows and the total length of arable and improved grassland field boundaries across England indicates that approximately 813,500 km of these boundaries (63% of them) are not currently hedgerows. While a small proportion of boundaries may be stone walls and ditches, the majority are likely to be delineated by fences and could thus be planted with hedgerows: a 40% increase in existing hedgerow length would amount to 193,415 km, or 11% of these un-hedged field boundaries being planted. Using our SOC_*ESM*_ sequestration rate of 1.48 Mg C ha^−1^ yr^−1^, and a hedgerow width of 1.5 m, we estimated that over 40 years 1.7 Tg C could be sequestered in the top 50 cm of soil beneath these new hedgerows. This is equivalent to the removal of 6.3 Tg of CO_2_ from the atmosphere over 40 years.

**Table 5:** Estimated existing hedgerow length (see 2.4.1), hedgerow planting goal set by the Climate Change Committee (Climate Change Committee, 2018) in England, and relative SOC sequestration potential after 40 years if the goals will be met.

Variable	Estimate	Unit
Existing hedgerows length	485,222	km
Hedgerows increment goal	40	%
Hedgerows increment length	193,415	km
Annual SOC seq. of increment	42,938	Mg C yr^−1^
Annual CO2 seq. of increment	157,583	Mg CO_2_ yr^−1^
SOC seq. in 40 years	1.7	Tg C
CO2 seq. in 40 years	6.3	Tg CO_2_

## 4 Discussion

Sequestration and storage of atmospheric C in agricultural soils has been identified as a necessary step towards meeting the Paris Climate Agreement goals of climate mitigation and woody linear features in farmed landscapes may contribute towards reaching these targets (Falloon et al., 2004; Schoeneberger et al., 2012; Axe et al., 2017). This study focused on estimating the role of hedgerows in SOC sequestration under hedges on five dairy farms in the Eden Valley, Cumbria, England. SOC stocks were higher beneath hedgerows than in adjacent fields, and we found that, according to ESM estimates, hedgerows stored in the top 50 cm an average of 164.0 (144.2–183.8) Mg C ha^−1^ compared to 124.9 (112.8–136.9) Mg C ha^−1^ in adjacent fields. We also showed that the SOC stock increased with hedgerow age and were therefore able to calculate the SOC sequestration rate associated with planting hedges in agricultural landscapes, which was estimated at 1.48 Mg C ha^−1^ for 37 years old hedgerows. We used this sequestration value to calculate SOC storage by 2050 beneath hedgerows planted within public and private AES in the Eden Valley and throughout England and estimated that a 40% increase in existing hedgerow length across England will correspond to 6.3 Tg of atmospheric CO_2_ being captured and stored in soil beneath hedgerows over the course of 40 years.

### 4.1 Soil organic carbon stocks beneath hedgerows

Our results show that SOC stocks beneath older hedges may be underestimated when not correcting for differences in soil mass beneath hedges and in adjacent fields. This supports the body of literature highlighting the importance of applying the ESM correction method when estimating SOC across different land use types (Murphy et al., 2004; VandenBygaart and Kay, 2004; Vero et al., 2014) instead of the traditional ESV methods used in most publications (Ellert and Bettany, 1995; VandenBygaart, 2006; Wendt and Hauser, 2013). The FD method has been shown to introduce bias when soil bulk density differs among treatments or has changed over time because of land use change (Lee et al., 2009; Schrumpf et al., 2011; Wendt and Hauser, 2013; Rovira et al., 2015; Juvinyà et al., 2021). SOC_*ESM*_ corrections have recently been advised as standard protocol by the Intergovernmental Panel on Climate Change (Ogle et al., 2019), thus, from this point on our discussion focuses on the SOC_*ESM*_ stocks presented in the results section.

Few studies have quantified SOC stocks beneath woody features in agricultural landscapes and comparisons among those that have can be challenging due to differences in hedgerow species, structure and management, climatic conditions, soil type and sampling depth. The average SOC stock under hedgerows at 0–50 cm depth in our study was 31% or 41.5 (27.2–55.9) Mg C ha^−1^ higher than in improved grasslands, a difference that increased to 49% or 57.5 (42.2–72.8) Mg C ha^−1^ greater when considering the 37 years old and Old hedgerows only. Comparatively, Cardinael et al. (2018a) reported a gain of 125 Mg C ha^−1^ in SOC stocks for an unspecified soil depth for cropland to hedgerow land use conversion, which is double what we observed for grassland to hedgerow land use change. Table 6 summarises relevant studies that measured SOC stocks at different depths under or close to linear woody features, from tree lines to frequently managed hedges in agricultural landscapes. As ours, these studies found that SOC stocks associated with hedgerows were higher than in adjacent agricultural fields. However, the magnitude of the stock varies greatly among them, partially reflecting differences in sampling depth. Other studies have derived SOC stock estimates from existing data; for example, in England, Robertson et al. (2012) conservatively estimated the SOC stock in the top 30 cm of soil beneath hedgerows to be 85 Mg C ha^−1^ based on figures derived from ancient woodland. Our SOC stock of 126.8 (1119–141.7) Mg C ha^−1^ for the same soil depth is close to their estimate. Moreover, our improved grassland field SOC stock of 97.3 (89.3–105.3) Mg C ha^−1^ in the top 30 cm, or 69.9 (65.3–74.5) in the top 20 cm, is comparable to that estimated by Chamberlain et al. (2010) of 67.2 Mg ha^−1^ in the top 15 cm for improved grassland in England. This suggests that the SOC stocks estimated in our study are in the same order of magnitude as others from improved grassland and beneath hedges in England.

**Table 6:** Comparison of SOC stock estimates beneath or close to hedgerows from European and North American studies.

Country	Publication	Koppen climate class	Soil type	Hedge/treeline spp.	Hedge age	Number of hedges	Sampling distance from hedge (m)	Depth sampled (cm)	Method	SOC stock (Mg C ha^−1^)	Our results (SOC_*ESM*_)
UK	Ford et al. (2019)	Temperate oceanic	Gleysols and Cambisols	*Prunus spinosa* L., *Crategus monogyna* Jacq.	10–40	82	1.5	0–15	SOC_*ESM*_	68	94.5 (80.6–108.4) 0–20 cm
UK	Ford et al. (2021)	Temperate oceanic	Gleysols and Cambisols	*Prunus spinosa* L., *Crategus monogyna* Jacq.	10–40	2	0.7	0–15	SOC_*ESM*_	41	
Belgium	Van Vooren et al. (2018)	Temperate oceanic	Cambisols	*Prunus spinosa* L., *Crategus monogyna* Jacq.	8–100	6	1	0–20	SOC_*FD*_	42	
Belgium	Van Den Berge et al. (2021)	Temperate	Arensol	*Betula pendula* Roth, *Quercus robur* L., *Prunus serotina* Ehrh.	58+	10	0	0–23	SOC_*FD*_	82	
Italy	Borin et al. (2010)	Humid subtropical	–	*Platanus hybrida* Brot., *Viburnum opulus* L.	2–20	83	0	0–5, 20–25	SOC_FD_*	83	
Canada	Amadi et al. (2016)	Cold continental	Chernazem	*Caragana arborescens* Lam., *Pinus sylvestris*	20	14	0	0–30	SOC_FD_*	106	126.8 (111.9–141.7) 0–30 cm
France	Viaud and Kunnemann (2021)	Temperate oceanic	Cambisol and Luvisols	*Quesrcus robur* L., *Carpinus betulus*, L. *Crategus monogyna* Jacq.	20–120	12	1	0–30	SOC_FD_*	85	
France	Walter et al. (2003)	Temperate oceanic	Arensol	*Bocage* hedgerows	10–40	6	2	0–50	SOC_FD_*	167	164.0 (144.2–183.8) 0–50 cm

The accumulation of SOC with hedgerow age shown by the increase in additional SOC (Fig. 2) is attributed to the belowground biomass growth and litter accumulation as hedgerows mature from recently planted saplings into fully grown plants (Fig. 1). SOC stocks beneath young hedgerows will depend on management associated with their planting, such as disturbance of the soil profile (Laganière et al., 2010) or the use of mulch (Chalker-Scott, 2007). Overall, 2–4 year old hedgerows showed SOC stock closer to that of fields than of older hedgerows (Table 3), despite all new hedgerows being planted over historical hedgerow boundaries. This shows that the removal of hedgerows results in the rapid loss of their associated SOC stocks, as found by Van Den Berge et al., (2021), and that this loss is on average 57.5 Mg C ha^−1^ for 1.5 m wide hedgerows older than 10 years. In England, it is estimated that 109,000 km of managed hedgerows were lost between 1984 and 2007 (Carey et al., 2007); if we assume this was associated with a loss of 57.5 Mg C ha^−1^ (8.6 Mg C km-1 for 1.5 m wide hedgerows) then this resulted in the loss of 0.94 Tg C (3.5 Tg CO_2_) from the soil in just over 20 years. The length of existing hedgerows in England (485,222 km) can thus be associated to the storage of 4.2 Tg C (15.3 Tg CO_2_) in the soil beneath them. These figures are based on the difference in SOC beneath mature hedges and grassland fields—in arable fields this is likely to be higher—and it illustrates how preserving existing hedgerows is just as important for climate change mitigation as planting new ones, as it ensures the persistence of SOC stored in the ground.

Differences in SOC stock at 0–30 and 0–50 cm depth highlight the importance of sampling to greater than the typical 0–15 or 0–30 cm depth when estimating SOC stocks under woody vegetation. The need for deeper sampling has been emphasised by others to avoid underestimating the capacity of trees and hedgerows to contribute towards climate mitigation (Nair, 2012; Harper and Tibbett, 2013; Yost and Hartemink, 2020). Roots of woody species, especially of shrubs, extend deeper into the soil than those of herbaceous species (Jackson et al., 1996) and input organic carbon through fine root turnover and exudates, contributing towards the accumulation of stable SOC in subsoils (Godbold et al., 2006; Rasse et al., 2006). Moreover, soils below 20 cm show a higher temporal residence of SOC due to reduced microbial activity, contributing to longer-term stabilisation and persistence of SOC in subsoil compared to surface soils (Fontaine et al., 2007; Rumpel and Kögel-Knabner, 2011). The IPCC recommends a minimum sampling depth of 30 cm for SOC stocks estimation (IPCC, 2006; Ogle et al., 2019), thus, most studies do not sample beyond the topsoil, with detrimental repercussions on SOC stock estimates (Nair, 2012; Van Vooren et al., 2018).

While Upson et al. (2016) found that below 40 cm trees did not show higher SOC stocks than agricultural fields, it is likely that differences in SOC may extend further into the subsoil than the 50 cm sampled in our study (Walter et al., 2003; Harper and Tibbett, 2013; Cardinael et al., 2015, 2017). For example, Viaud and Kunnemann (2021) used bulk density estimates calculated from pedotransfer functions to estimate the SOC stock down to a sampling depth of 90 cm and found that >20 year old bocage hedgerows had significantly higher SOC stocks than adjacent fields. Using these functions, however, is usually not advised when estimating SOC stocks as it can underestimate their variance (Schrumpf et al., 2011). Similarly, Amadi et al. (2016) found that the average SOC stock at 0–100 cm depth was higher beneath shelterbelt vegetation than in adjacent cropped fields.

Our SOC stocks estimates can be used to calculate overall C storage by hedgerows of similar characteristics and species composition. Axe et al. (2017) attributed hedge height as the main driving force of aboveground carbon stock and showed that 1.9 m tall hawthorn and blackthorn hedgerow biomass stored 32.2 Mg C ha^−1^ aboveground and 38.2 Mg C ha^−1^ belowground. The 37 years old and Old hedgerows investigated in our study were of comparable dimensions and species composition, suggesting that C storage in their aboveground and belowground biomass (70.4 Mg C ha^−1^) may amount to a third of their SOC stock at 0–50 cm depth (185.5 Mg C ha^−1^). Therefore, according to Axe et al. (2017) biomass C storage estimate and our SOC stock estimate, the total C storage associated with mature hedgerows dominated by these species is 255.9 Mg C ha^−1^, or 38.5 Mg C km (assuming a hedgerow width of 1.5 m and a soil depth of 0–50 cm).

### 4.2 SOC sequestration by hedgerows

This is the first study to quantify the SOC sequestration rate associated with planting hedgerows, which we estimated to be 1.48 Mg C ha^−1^ yr^−1^ at 0–50 cm depth for 37 year old hedgerows, 13% higher than the SOC sequestration rate obtained from SOC_*FD*_ measures. Although it is often stated that woody features in agricultural landscapes can sequester substantial amounts of SOC (Schoeneberger et al., 2012), few studies have attempted to estimate their SOC sequestration capacity and most of these have focused on patches of trees. For example, in the United States, Hernandez-Ramirez et al. (2011) calculated sequestration rates of 0.11 Mg C ha^−1^ yr^−1^ in the top 15 cm of soils beneath 35 years old coniferous afforestation sites, while Sauer et al. (2012) assessed the sequestration rate for coniferous plantations and native hardwoods to be 0.56 Mg C ha^−1^ yr^−1^ at 0–30 cm depth. In England, Crossland (2015) is among the few to suggest SOC sequestration rates associated with planting hedgerows, modelled from a small number of field observations, of 2.7–12.2 Mg C ha^−1^ yr^−1^. These figures, however, were based on unmanaged hedgerows and are closer to SOC sequestration rates associated with agroforestry systems such as intercropping (Lorenz and Lal, 2014). Also in England, based on data from Rothamsted woodland data, Robertson et al. (2012) reported a SOC sequestration potential estimate of 0.46 Mg C ha^−1^ yr^−1^ in the top 30 cm of soil beneath mature hedgerows. Our sequestration rate, which is accounting for changes in bulk density between fields and hedges, is twice as much, indicating a substantially higher SOC sequestration potential of regularly managed hedgerows than previously thought.

The capacity of soils to sequester carbon is finite and soils will reach equilibrium depending on the quantity and quality of organic inputs (Caruso et al., 2018) and soil properties; therefore, SOC sequestration beneath hedgerows will not continue indefinitely. For example, Falloon et al. (2004) assumed that it would take between 50 and 100 years for soil carbon to reach a new equilibrium following a land use change. Agroforestry research indicates that woody vegetation in agricultural landscapes can continue to sequester C for decades (Hernandez-Ramirez et al., 2011; Poeplau et al., 2011), while long-term woodland data suggests that soils may continue to accumulate SOC for centuries (Wolton et al., 2014). Reduced wind and water erosion, absence of cultivation practices and management disturbances compared to adjacent fields, together with plant species diversity are likely contributors to hedgerows’ increased stability of SOC stocks and prolonged SOC sequestration capacity (Schoeneberger et al., 2012; Lorenz and Lal, 2014; Thiel et al., 2015; Ford et al., 2019).

The capacity of hedges to sequester C is not limited to soil sequestration, as C also accumulates in their aboveground and belowground biomass, albeit to a lower degree than within soil. Borin et al. (2010) estimated that buffer strips of alternating fast-growing trees and large shrubs may store up to 21.8 Mg C ha^−1^ yr^−1^ within soil and their total biomass when compared to adjacent arable fields, while a recent meta-analysis by Drexler et al. (2021) estimated that the establishment of hedgerows on cropland could sequester between 2.1 and 5.2 Mg C ha^−1^ yr^−1^ in their biomass and soil for a period of 50 and 20 years, respectively. Cardinael et al. (2018b), instead, estimated C sequestration rates of 0.87 Mg C ha yr^−1^ in aboveground biomass and 0.23 Mg C ha yr^−1^ in belowground biomass for hedgerows in temperate climates. In the UK, where 70% of hedges are 1–2 m wide and ∼60% are 1–2 m high (Barr and Gillespie, 2000), regular trimming hinders total biomass growth, and sequestration estimates are around 1 Mg C ha^−1^ yr^−1^. For example, Falloon et al. (2004) estimated from Rothamsted woodland data an aboveground accumulation potential of 1.0 Mg ha^−1^ yr^−1^, and Kay et al. (2018) estimated it as 0.3–0.75 Mg C ha^−1^ yr^−1^ for aboveground and belowground biomass together. Recently, Blair (2021) measured an average aboveground biomass sequestration of 1.2 Mg C ha^−1^ yr^−1^ for intensively managed hedgerows. Using these figures, Table 7 presents hedgerows sequestration estimates in SOC and aboveground biomass for hedgerows of different widths, assuming that wide hedgerows maintain the same shrub density prescribed within AES of at least nine shrubs per meter in staggered rows (Wolton et al., 2013).

**Table 7:** Estimated annual SOC_*ESM*_ sequestration rates of hedgerows assuming a hedgerow width of 1.5 m and of 2 m at 0–50 depth of the soil profile based on 37 years old hedgerows. Estimates for SOC and aboveground biomass (AGB) are shown, with ABG values of 1 Mg C ha^−1^ yr^−1^ derived from Falloon et al. (2004). Estimates for 2 m wide hedgerows assume the same shrub density prescribed for 1.5 m wide hedgerows.

Component	Hedge width (m)	Mg C ha^−1^ yr^−1^	Mg C km^−1^ yr^−1^	Mg CO_2_ km^−1^ yr^−1^
SOC	1.5	1.48	0.22	0.81
	2		0.30	1.09
SOC + AGB	1.5	2.48	0.37	1.37
	2		0.50	1.82

### 4.3 The climate change mitigation potential of planting hedgerows

Strong emphasis has been put on the role of agricultural landscapes for climate mitigation goals (Schoeneberger et al., 2012; Frank et al., 2017; Styles et al., 2018), as well as on the multi-functional delivery of ecosystem services by hedgerows (Wehling and Diekmann, 2009; Staley et al., 2012; Montgomery et al., 2020; Viaud and Kunnemann, 2021). Our findings show that, as hedgerows mature, SOC stocks progressively accumulate in the soil beneath them over several decades. Therefore, our results indicate that hedgerow planting may be used for C sequestration in agricultural landscapes. In the UK, Falloon et al. (2004) calculated from SOC sequestration rates of natural woodland regeneration that the SOC storage contribution after 50 years of planting ∼79,000 km of vegetated field boundaries comprising hedgerows and associated grass strips would range 0.1–1.2% of the UK's agricultural annual CO_2_ emissions. Today, in England, CO_2_ emissions from agriculture are estimated at 5.6 Tg CO_2_ yr^−1^ (DEFRA, 2019); thus, based on our findings, by 2050 the soil beneath hedgerows planted within AES between 2004 and 2019 around improved grassland and arable fields will have sequestered 0.85% of 5.6 Tg CO_2_, which equates on average to just 0.02% of annual CO_2_ emissions from agriculture a year.

If the goal set by the Climate Change Committee to increase total hedgerow length by 40% in England will be met, in 40 years the accumulation of SOC stock beneath these newly planted hedgerows would sequester 6.3 Tg CO_2_. Over half (∼55%) of this planting goal will compensate the loss of managed hedgerows in England between 1984 and 2007 (Carey et al., 2007) and their associated SOC stocks; nonetheless, increasing existing hedgerow length by 40% will annually offset 2.81% of CO_2_ emissions from agriculture for four decades. We can also consider the C sequestration rates shown in Table 7 that account for hedgerow biomass and soil together, as well as for a marginal increase in hedgerow width. Then, these figures would result in 4.72% and 6.29% of annual agricultural CO_2_ emissions being offset for 40 years, respectively, as a result of planting hedgerows. These figures likely underestimate to some degree the sequestration potential of hedgerows, as we have only quantified SOC stocks to a depth of 50 cm. Nonetheless, our results indicate a substantial capacity of hedgerows to capture and store anthropogenic CO_2_ emissions in agricultural landscapes if we were to increase hedgerow length by 40%. However, to meet this goal, hedge planting in England needs to increase. Over the period 2004–2019 in England, AES have planted the equivalent of only 1.5% of the total hedgerows lost in 1984–2007. At their highest rate yet (424 km yr^−1^ in 2019) it would take 455 years to achieve the 40% increase in hedgerow length in intensive agricultural landscapes. Although planting efforts within these schemes have increased over time, these are not feasible time frames to benefit from the climate change mitigation potential of hedgerows and reach net-zero goals by 2050. Indubitably, hedgerow planting within AES does not reflect the entirety of new hedges planted in England during this period. Private sector initiatives, such as the Milk Plan scheme, and farmers’ initiative will have also contributed towards further hedgerow expansion; however, it is not possible to quantify the extent of this contribution.

Based on our annual C sequestration rate of 1.48 Mg C ha^−1^ yr^−1^, hedgerows planted within AES and the Milk Plan scheme will sequester similar amounts of SOC by 2050 in the Eden Valley. This suggests comparable results of public and private initiatives for hedgerow planting in the region, with the Milk Plan reaching analogous results to AES over a shorter time frame (three years compared to 15). In England, there were an estimated 102,969 agricultural holdings in 2015 (DEFRA, 2016). Replicating across the country Milk Plan's hedgerow planting rate, which was 3.2 (2.5–3.9) km yr^−1^ across twenty-seven farms, would see England's planting rate raising to 12,204 km yr^−1^. This would allow the Climate Change Committee (2018) goal of 40% more hedgerows to be achieved in just 16 years, vastly faster than the centuries required at current AES rates. Thus, planting rates need to increase dramatically across England if we are to benefit from the climate change mitigation potential of hedgerow planting.

A considerable change is needed to incentivise hedgerow planting rates within agricultural landscapes in England to meet the goal set by the Climate Change Committee. This could be achieved via several mechanisms, such as (i) increasing payments in AES for the delivery of public goods as well as compensating for costs and time of implementation and management, (ii) harnessing private sector funding, and (iii) allowing farmers to sell carbon credits in private markets (Climate Change Committee, 2020a; Reed et al., 2020). The high planting rates achieved by the Milk Plan in the Eden Valley can be related largely to the reduced number of options offered within the scheme, the simplified evaluation process, and the flexibility in the physical implementation of the options on the farm (Coyne et al., 2021). Harnessing the investment of the private sector will likely be essential in upscaling these results nationally, either with individual private supply chain schemes, such as the Milk Plan, which tie hedgerow planting to a guaranteed price for product, or with collaborative schemes models of collaborations between public and private sector initiatives (e.g. Landscape Enterprise Networks, Gosal et al., 2020). If hedgerow planting can be encouraged widely, hedgerows will be a valuable tool for atmospheric C sequestration and storage, making a significant contribution to climate change mitigation targets and net-zero 2050 goals.

We have used the SOC sequestration rate of 37 years old hedgerows to upscale our results over a 40 years period, as beyond this we are likely to overestimate their ability to sequester carbon. This sequestration rate may not be representative of all hedgerows, as the rate of SOC storage is influenced by external factors of soil type and climatic conditions, as well as hedgerow structure and species composition (Thiel et al., 2015; Ford et al., 2021). Rainfall water accumulation and discharge, for example, affect erosion rates and both high and low soil moisture affect soil SOC storage by reducing microbial activity (Frank et al., 2015). While the capacity of hedgerows to sequester carbon will vary depending on their environment and management, English improved grassland landscapes are concentrated in similar climatic areas, rendering our estimation suitable for climate mitigation modelling in England. It should be noted that we have used the same SOC sequestration estimate for hedgerows planted around improved grassland and arable fields. However, arable soils typically have lower SOC stocks than grassland soils, and studies of agroforestry and afforestation establishment on grassland usually show smaller SOC stock changes than for cropland (Ogle et al., 2019). Further research is required to determine if this is also the case for planting hedgerows on arable soils or semi-natural grassland, as well as to assess C sequestration rates in the biomass of managed hedgerows (Drexler et al., 2021).

Atmospheric C sequestration and storage in soils cannot be the only agricultural contribution towards climate change mitigation, as GHG emissions reduction and land-use changes also need to be addressed (Powlson et al., 2011). Although hedgerow planting alone will not allow farms to reach agricultural net-zero targets by 2050, our results indicate that hedgerows, together with their supporting and provisioning ecosystem services, may also be used as a means for atmospheric CO_2_ sequestration.

## Credit author statement

Author contributions SB Data curation, Formal analysis; Writing -Original draft; Writing -Review and editing. PJC Conceptualization; Methodology; Writing -Review and editing, Supervision. RPG Methodology, Data curation; Writing -Review and editing. GZ Conceptualization, Writing-Review and editing, Project administration, Funding acquisition.

## Declaration of competing interest

The authors declare that they have no known competing financial interests or personal relationships that could have appeared to influence the work reported in this paper.

## Figures and Tables

**Figure A.1:**
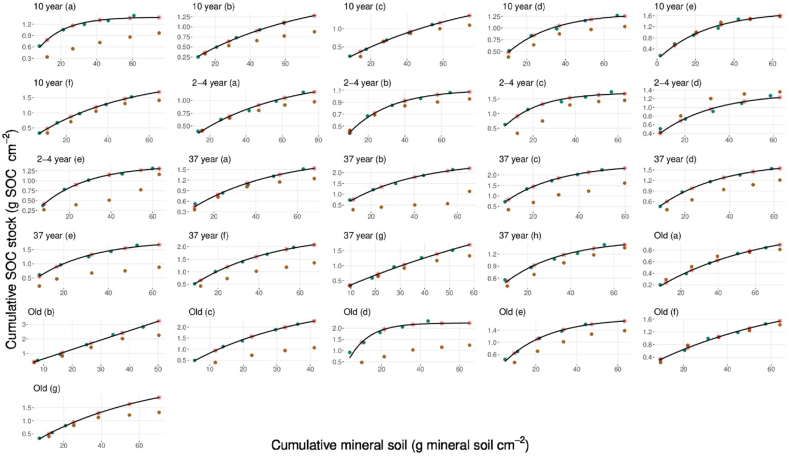
Graphical representation of the cumulative equivalent soil mass profiles in the study. Following a cumulative coordinates approach, the hedgerow soil organic carbon (SOC_*ESM*_) stocks represented by the red asterisks are obtained by interpolating the hedgerow measurements (green points) at the field reference cumulative mineral soil masses (brown points). The interpolation curve is defined in Equation (2). (For interpretation of the references to colour in this figure legend, the reader is referred to the Web version of this article.)

**Table B.1:** Estimated regression parameters, standard errors, t-statistic, and significance of the linear models of SOC stock estimates across all sampling depths (0–50 cm). C = cambisol, S = stagnosol. *P < 0.05; **P < 0.01; ***P < 0.001.

	Variable	Estimate	SE	t-Statistics	Sig.
SOC_*FD*_	Intercept	2.84	0.05	57.13	***
	2–4 years	0.19	0.08	2.32	*
	10 years	0.20	0.08	2.50	*
	37 years	0.40	0.07	5.41	***
	Old	0.31	0.08	3.98	***
	Depth (cm)	−0.37	0.02	−15.63	***
	Rainfall (mm)	0.08	0.03	2.59	*
	Temperature C	0.12	0.03	3.88	***
	C vs S	0.33	0.06	5.86	***
SOC_*ESM*_	Intercept	4.29	0.14	29.84	***
	2–4 years	0.20	0.24	0.81	ns
	10 years	0.46	0.23	2.01	*
	37 years	0.97	0.21	4.59	***
	Old	0.84	0.23	3.74	***
	Depth (cm)	−1.11	0.07	−16.17	***
	Rainfall (mm)	0.12	0.09	1.40	ns
	Temperature C	0.34	0.09	3.76	***
	C vs S	0.80	0.16	4.88	***
